# 4-Hy­droxy-3-meth­oxy­benzaldehyde thio­semicarbazone

**DOI:** 10.1107/S1600536813032303

**Published:** 2013-11-30

**Authors:** Adriano Bof de Oliveira, Bárbara Regina Santos Feitosa, Christian Näther, Inke Jess

**Affiliations:** a Departamento de Química, Universidade Federal de Sergipe, Av. Marechal Rondon s/n, Campus, 49100-000 São Cristóvão–SE, Brazil; b Institut für Anorganische Chemie, Christian-Albrechts-Universität zu Kiel, Max-Eyth Strasse 2, D-24118 Kiel, Germany

## Abstract

In the title compound, C_9_H_11_N_3_S, there is an intra­molecular O—H⋯O hydrogen bond involving the OH group and the adjacent methoxy O atom. The mol­ecule is essentially planar, with the maximum deviation from the mean plane of the non-H atoms being 0.1127 (14) Å for the methyl C atom. In the crystal, mol­ecules are connected *via* centrosymmetric pairs of N—H⋯S and O—H⋯O hydrogen bonds into a two-dimensional network parallel to (10-3).

## Related literature

For the *in vitro* anti­malarial and anti­tubercular activity of hy­droxy-meth­oxy­benzaldehyde thio­semicarbazone derivatives, see: Khanye *et al.* (2011 ▶). For the first report of the synthesis, see: Freund & Schander (1902 ▶). For the synthesis and crystal structure of an isomer of the title compound, see: Hao (2010 ▶).
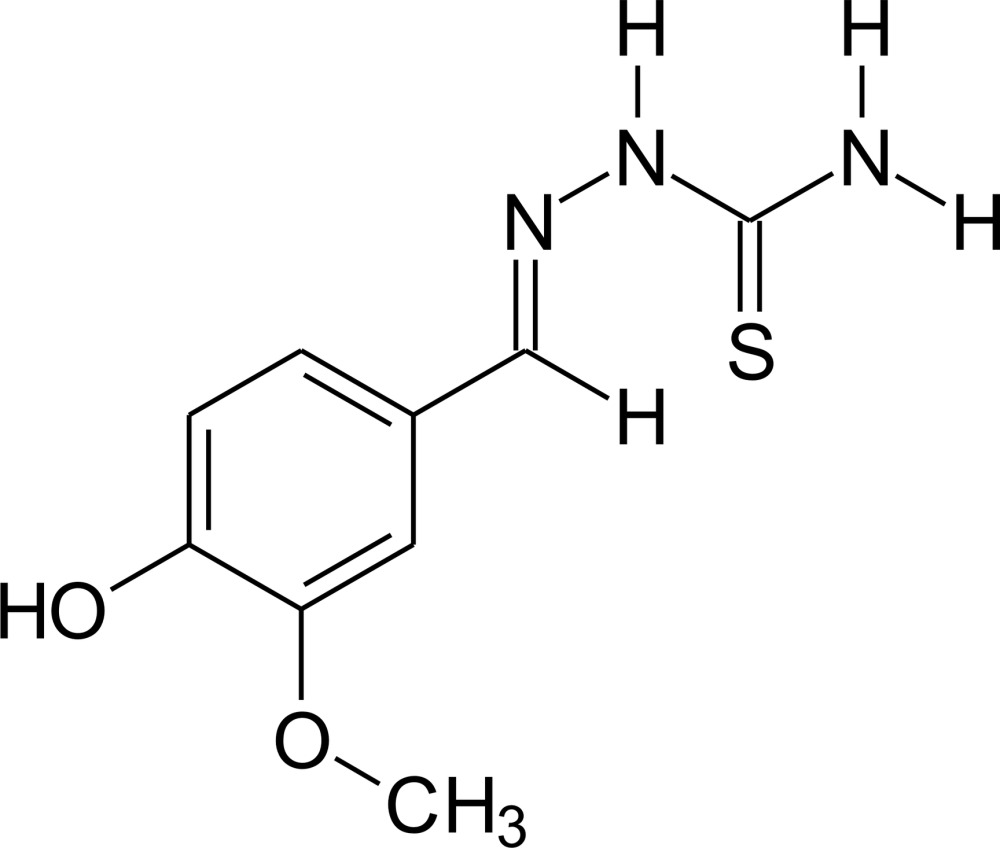


## Experimental

### 

#### Crystal data

C_9_H_11_N_3_O_2_S
*M* *_r_* = 225.27
Triclinic, 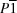

*a* = 4.5886 (5) Å
*b* = 8.5213 (11) Å
*c* = 13.9621 (15) Å
α = 75.898 (13)°
β = 87.669 (13)°
γ = 77.580 (14)°
*V* = 517.05 (10) Å^3^
*Z* = 2
Mo *K*α radiation
μ = 0.30 mm^−1^
*T* = 200 K
0.4 × 0.3 × 0.2 mm

#### Data collection

Stoe IPDS-1 diffractometer
Absorption correction: numerical (*X-SHAPE* and *X-RED32*; Stoe & Cie, 2008 ▶) *T*_min_ = 0.865, *T*_max_ = 0.982
5179 measured reflections
2211 independent reflections
1829 reflections with *I* > 2σ(*I*)
*R*_int_ = 0.040

#### Refinement

*R*[*F* ^2^ > 2σ(*F* ^2^)] = 0.036
*wR*(*F* ^2^) = 0.100
*S* = 1.03
2211 reflections
139 parameters
H-atom parameters constrained
Δρ_max_ = 0.32 e Å^−3^
Δρ_min_ = −0.27 e Å^−3^

### 

Data collection: *X-AREA* (Stoe & Cie, 2008 ▶); cell refinement: *X-AREA*; data reduction: *X-RED32* (Stoe & Cie, 2008 ▶); program(s) used to solve structure: *SHELXS97* (Sheldrick, 2008 ▶); program(s) used to refine structure: *SHELXL97* (Sheldrick, 2008 ▶); molecular graphics: *DIAMOND* (Brandenburg, 2006 ▶); software used to prepare material for publication: *publCIF* (Westrip, 2010 ▶).

## Supplementary Material

Crystal structure: contains datablock(s) I. DOI: 10.1107/S1600536813032303/fy2108sup1.cif

Structure factors: contains datablock(s) I. DOI: 10.1107/S1600536813032303/fy2108Isup2.hkl

Supplementary material file. DOI: 10.1107/S1600536813032303/fy2108Isup3.cml

Additional supplementary materials:  crystallographic information; 3D view; checkCIF report

## Figures and Tables

**Table 1:** Hydrogen-bond geometry (Å, °)

*D*—H⋯*A*	*D*—H	H⋯*A*	*D*⋯*A*	*D*—H⋯*A*
O1—H1⋯O2	0.84	2.24	2.6934 (16)	114
O1—H1⋯O2^i^	0.84	2.27	2.9153 (15)	134
N2—H2*A*⋯S1^ii^	0.88	2.59	3.4319 (14)	161
N3—H3*B*⋯S1^iii^	0.88	2.59	3.4540 (15)	169

Symmetry codes: (i) 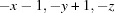; (ii) 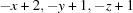; (iii) 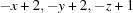.

**Fig. 1.**
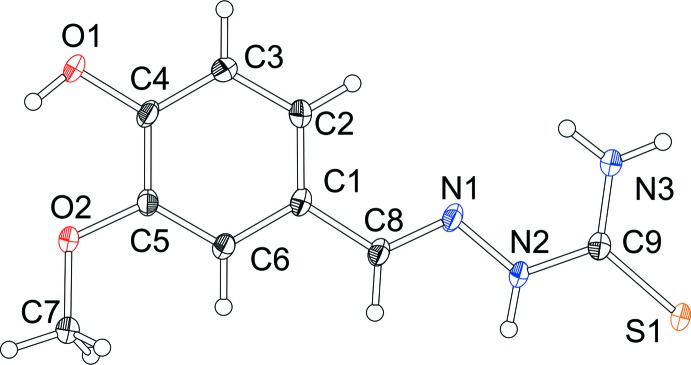
Molecular structure of the title compound with labeling and displacement ellipsoids drawn at the 70% probability level.

**Fig. 2.**
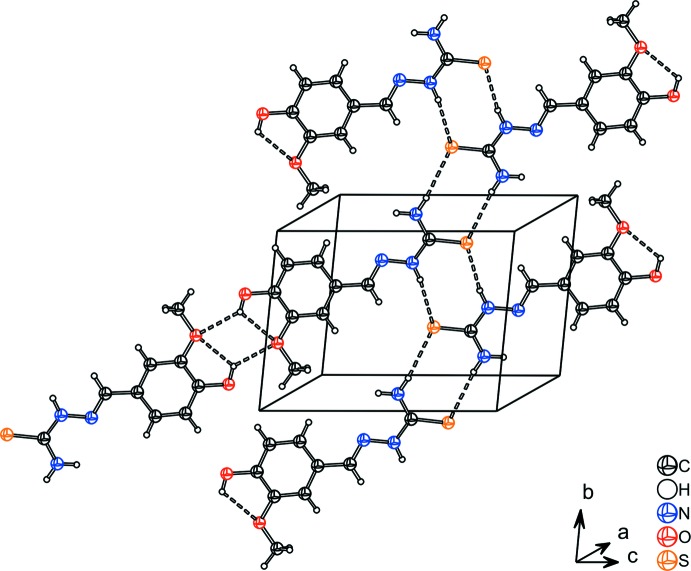
Crystal structure of the title compound with hydrogen bonds shown as dashed lines.

## supplementary crystallographic information

### 1. Comment

Thiosemicarbazone derivatives have a wide range of pharmacological properties. For example, benzaldehyde-thiosemicarbazone derivatives show *in vitro* antimalarial and antitubercular activity (Khanye *et al.*, 2011). As part of our study on the synthesis of thiosemicarbazone derivatives, we report herein the crystal structure of 4-hydroxy-3-methoxybenzaldehyde thiosemicarbazone. In the title compound (Fig. 1), in which the molecular structure matches the asymmetric unit, the maximal deviation from the least squares plane through all non-hydrogen atoms amount to 0.1127 (14) Å for C7. The molecule shows a *trans* conformation for the atoms about the C8—N1/N1—N2/N2—C9/ bonds. This conformation is also observed in the literature for an isomer of the title compound (Hao, 2010). The mean deviations from the least squares planes for the C1—C8/O1—O2 and C9/N1—N3/S1 fragments amount to 0.0733 (12) Å for C7 and 0.0188 (10) Å for N2, respectively, and the dihedral angle between the two planes is 5.08 (6)°.

The molecules are connected *via* centrosymmetric pairs of N—H···S and O—H···O hydrogen interactions, forming a two-dimensional H-bonded polymer. An O—H···O intramolecular H-interaction is also observed (Fig. 2 and Table 1). The molecules are arranged in layers, stacked along the *a-*axis direction through π–π-interactions, with the shortest C···C distance being 3.380 (23) Å [C8···C5^iv^, (iv): 1+*x*, *y*, *z*].

### 2. Experimental

The starting materials were commercially available and were used without further purification. The 4-hydroxy-3-methoxybenzaldehyde thiosemicarbazone synthesis was adapted from a procedure reported previously (Freund & Schander, 1902). The hydrochloric acid catalyzed reaction of vanillin (8.83 mmol) and thiosemicarbazide (8.83 mmol) in ethanol (50 ml) was refluxed for 6 h. After cooling and filtering, the title compound was obtained. Crystals suitable for X-ray diffraction were obtained from the reaction mixture by the slow evaporation of solvent.

### 3. Refinement

All H atoms were were positioned with idealized geometry (methyl and O—H H atoms allowed to rotate but not to tip) and were refined as isotropic with *U*_iso_(H) = 1.2 or 1.5 *U*_eq_(C, N and O) using a riding model with C—H = 0.95 Å for aromatic H atoms, C—H = 0.98 for methyl H atoms, N—H = 0.88 Å for amine and hydrazine H atoms and O—H = 0.84 Å for the O—H H atom.

### Crystal data

**Table:**

C_9_H_11_N_3_O_2_S	*Z* = 2
*M**_r_* = 225.27	*F*(000) = 236
Triclinic, *P*1	*D*_x_ = 1.447 Mg m^−^^3^
Hall symbol: -P 1	Mo *K*α radiation, λ = 0.71073 Å
*a* = 4.5886 (5) Å	Cell parameters from 5179 reflections
*b* = 8.5213 (11) Å	θ = 2.5–27.0°
*c* = 13.9621 (15) Å	µ = 0.30 mm^−^^1^
α = 75.898 (13)°	*T* = 200 K
β = 87.669 (13)°	Block, yellow
γ = 77.580 (14)°	0.4 × 0.3 × 0.2 mm
*V* = 517.05 (10) Å^3^	

### Data collection

**Table:**

Stoe IPDS-1 diffractometer	2211 independent reflections
Radiation source: fine-focus sealed tube, Stoe IPDS-1	1829 reflections with *I* > 2σ(*I*)
Graphite monochromator	*R*_int_ = 0.040
φ scans	θ_max_ = 27.0°, θ_min_ = 2.5°
Absorption correction: numerical (*X-SHAPE* and *X-RED32*; Stoe & Cie, 2008)	*h* = −5→5
*T*_min_ = 0.865, *T*_max_ = 0.982	*k* = −10→10
5179 measured reflections	*l* = −17→17

### Refinement

**Table:**

Refinement on *F*^2^	Secondary atom site location: difference Fourier map
Least-squares matrix: full	Hydrogen site location: inferred from neighbouring sites
*R*[*F*^2^ > 2σ(*F*^2^)] = 0.036	H-atom parameters constrained
*wR*(*F*^2^) = 0.100	*w* = 1/[σ^2^(*F*_o_^2^) + (0.0666*P*)^2^] where *P* = (*F*_o_^2^ + 2*F*_c_^2^)/3
*S* = 1.03	(Δ/σ)_max_ = 0.001
2211 reflections	Δρ_max_ = 0.32 e Å^−^^3^
139 parameters	Δρ_min_ = −0.27 e Å^−^^3^
0 restraints	Extinction correction: *SHELXL97* (Sheldrick, 2008), Fc^*^=kFc[1+0.001xFc^2^λ^3^/sin(2θ)]^-1/4^
Primary atom site location: structure-invariant direct methods	Extinction coefficient: 0.055 (15)

### Special details

**Table:**

Geometry. All e.s.d.'s (except the e.s.d. in the dihedral angle between two l.s. planes) are estimated using the full covariance matrix. The cell e.s.d.'s are taken into account individually in the estimation of e.s.d.'s in distances, angles and torsion angles; correlations between e.s.d.'s in cell parameters are only used when they are defined by crystal symmetry. An approximate (isotropic) treatment of cell e.s.d.'s is used for estimating e.s.d.'s involving l.s. planes.
Refinement. Refinement of *F*^2^ against ALL reflections. The weighted *R*-factor *wR* and goodness of fit *S* are based on *F*^2^, conventional *R*-factors *R* are based on *F*, with *F* set to zero for negative *F*^2^. The threshold expression of *F*^2^ > σ(*F*^2^) is used only for calculating *R*-factors(gt) *etc*. and is not relevant to the choice of reflections for refinement. *R*-factors based on *F*^2^ are statistically about twice as large as those based on *F*, and *R*- factors based on ALL data will be even larger.

### Fractional atomic coordinates and isotropic or equivalent isotropic displacement parameters (Å^2^)

**Table:**

	*x*	*y*	*z*	*U*_iso_*/*U*_eq_	
C1	0.1779 (3)	0.65909 (18)	0.22576 (11)	0.0210 (3)	
C2	0.0643 (3)	0.81737 (19)	0.16881 (12)	0.0252 (3)	
H2	0.1261	0.9096	0.1820	0.030*	
C3	−0.1384 (4)	0.84153 (19)	0.09296 (12)	0.0255 (3)	
H3	−0.2163	0.9501	0.0546	0.031*	
C4	−0.2278 (3)	0.70678 (19)	0.07295 (11)	0.0223 (3)	
C5	−0.1165 (3)	0.54775 (17)	0.12986 (11)	0.0203 (3)	
C6	0.0858 (3)	0.52373 (18)	0.20585 (11)	0.0207 (3)	
H6	0.1622	0.4152	0.2445	0.025*	
O1	−0.4253 (3)	0.73483 (14)	−0.00291 (9)	0.0306 (3)	
H1	−0.4773	0.6463	−0.0034	0.046*	
O2	−0.2264 (3)	0.42408 (13)	0.10546 (8)	0.0268 (3)	
C7	−0.1404 (4)	0.2626 (2)	0.16928 (14)	0.0354 (4)	
H7A	0.0761	0.2238	0.1664	0.053*	
H7B	−0.2414	0.1863	0.1481	0.053*	
H7C	−0.1969	0.2671	0.2372	0.053*	
C8	0.3893 (3)	0.62554 (19)	0.30740 (11)	0.0218 (3)	
H8	0.4565	0.5145	0.3439	0.026*	
N1	0.4870 (3)	0.74084 (16)	0.33129 (9)	0.0222 (3)	
N2	0.6853 (3)	0.68715 (15)	0.40998 (10)	0.0222 (3)	
H2A	0.7455	0.5805	0.4362	0.027*	
C9	0.7865 (3)	0.79732 (18)	0.44648 (11)	0.0218 (3)	
N3	0.6948 (4)	0.95590 (17)	0.40253 (11)	0.0344 (4)	
H3A	0.5720	0.9854	0.3514	0.041*	
H3B	0.7565	1.0318	0.4244	0.041*	
S1	1.01872 (9)	0.72984 (5)	0.54636 (3)	0.02745 (16)	

### Atomic displacement parameters (Å^2^)

**Table:**

	*U*^11^	*U*^22^	*U*^33^	*U*^12^	*U*^13^	*U*^23^
C1	0.0178 (7)	0.0265 (7)	0.0203 (7)	−0.0054 (6)	−0.0038 (5)	−0.0074 (6)
C2	0.0253 (8)	0.0239 (7)	0.0286 (8)	−0.0074 (6)	−0.0071 (6)	−0.0074 (6)
C3	0.0270 (8)	0.0216 (7)	0.0265 (8)	−0.0051 (6)	−0.0080 (6)	−0.0015 (6)
C4	0.0207 (7)	0.0270 (7)	0.0193 (7)	−0.0040 (6)	−0.0073 (6)	−0.0054 (6)
C5	0.0203 (7)	0.0223 (7)	0.0208 (7)	−0.0055 (6)	−0.0027 (5)	−0.0085 (6)
C6	0.0203 (7)	0.0229 (7)	0.0193 (7)	−0.0041 (6)	−0.0040 (6)	−0.0054 (6)
O1	0.0350 (6)	0.0285 (6)	0.0283 (6)	−0.0066 (5)	−0.0196 (5)	−0.0035 (5)
O2	0.0334 (6)	0.0224 (5)	0.0266 (6)	−0.0065 (4)	−0.0144 (5)	−0.0064 (4)
C7	0.0458 (10)	0.0233 (8)	0.0377 (10)	−0.0093 (7)	−0.0219 (8)	−0.0031 (7)
C8	0.0192 (7)	0.0261 (7)	0.0212 (7)	−0.0049 (5)	−0.0050 (6)	−0.0070 (6)
N1	0.0197 (6)	0.0278 (6)	0.0204 (6)	−0.0038 (5)	−0.0070 (5)	−0.0078 (5)
N2	0.0232 (6)	0.0224 (6)	0.0224 (7)	−0.0045 (5)	−0.0095 (5)	−0.0068 (5)
C9	0.0210 (7)	0.0247 (7)	0.0217 (7)	−0.0060 (6)	−0.0020 (6)	−0.0080 (6)
N3	0.0470 (9)	0.0227 (7)	0.0346 (8)	−0.0065 (6)	−0.0228 (7)	−0.0059 (6)
S1	0.0345 (3)	0.0250 (2)	0.0248 (2)	−0.00760 (16)	−0.01444 (16)	−0.00630 (15)

### Geometric parameters (Å, º)

**Table:**

C1—C2	1.390 (2)	O2—C7	1.428 (2)
C1—C6	1.401 (2)	C7—H7A	0.9800
C1—C8	1.4608 (19)	C7—H7B	0.9800
C2—C3	1.386 (2)	C7—H7C	0.9800
C2—H2	0.9500	C8—N1	1.2801 (19)
C3—C4	1.390 (2)	C8—H8	0.9500
C3—H3	0.9500	N1—N2	1.3792 (17)
C4—O1	1.3643 (17)	N2—C9	1.3404 (19)
C4—C5	1.393 (2)	N2—H2A	0.8800
C5—O2	1.3779 (17)	C9—N3	1.324 (2)
C5—C6	1.386 (2)	C9—S1	1.6962 (15)
C6—H6	0.9500	N3—H3A	0.8800
O1—H1	0.8400	N3—H3B	0.8800
			
C2—C1—C6	119.42 (13)	O2—C7—H7A	109.5
C2—C1—C8	123.13 (13)	O2—C7—H7B	109.5
C6—C1—C8	117.45 (13)	H7A—C7—H7B	109.5
C3—C2—C1	120.45 (13)	O2—C7—H7C	109.5
C3—C2—H2	119.8	H7A—C7—H7C	109.5
C1—C2—H2	119.8	H7B—C7—H7C	109.5
C2—C3—C4	119.99 (14)	N1—C8—C1	122.15 (14)
C2—C3—H3	120.0	N1—C8—H8	118.9
C4—C3—H3	120.0	C1—C8—H8	118.9
O1—C4—C3	118.45 (13)	C8—N1—N2	114.48 (12)
O1—C4—C5	121.52 (13)	C9—N2—N1	120.04 (12)
C3—C4—C5	120.02 (13)	C9—N2—H2A	120.0
O2—C5—C6	124.88 (13)	N1—N2—H2A	120.0
O2—C5—C4	115.17 (13)	N3—C9—N2	117.44 (14)
C6—C5—C4	119.95 (13)	N3—C9—S1	123.06 (11)
C5—C6—C1	120.17 (14)	N2—C9—S1	119.49 (11)
C5—C6—H6	119.9	C9—N3—H3A	120.0
C1—C6—H6	119.9	C9—N3—H3B	120.0
C4—O1—H1	109.5	H3A—N3—H3B	120.0
C5—O2—C7	116.19 (11)		
			
C6—C1—C2—C3	0.1 (2)	C2—C1—C6—C5	0.1 (2)
C8—C1—C2—C3	−179.27 (15)	C8—C1—C6—C5	179.45 (13)
C1—C2—C3—C4	−0.5 (3)	C6—C5—O2—C7	5.2 (2)
C2—C3—C4—O1	−179.32 (15)	C4—C5—O2—C7	−173.55 (15)
C2—C3—C4—C5	0.8 (3)	C2—C1—C8—N1	−0.7 (2)
O1—C4—C5—O2	−1.7 (2)	C6—C1—C8—N1	179.91 (14)
C3—C4—C5—O2	178.21 (14)	C1—C8—N1—N2	−179.89 (13)
O1—C4—C5—C6	179.46 (14)	C8—N1—N2—C9	−175.18 (14)
C3—C4—C5—C6	−0.6 (2)	N1—N2—C9—N3	−1.7 (2)
O2—C5—C6—C1	−178.53 (14)	N1—N2—C9—S1	177.37 (11)
C4—C5—C6—C1	0.2 (2)		

### Hydrogen-bond geometry (Å, º)

**Table:**

*D*—H···*A*	*D*—H	H···*A*	*D*···*A*	*D*—H···*A*
O1—H1···O2	0.84	2.24	2.6934 (16)	114
O1—H1···O2^i^	0.84	2.27	2.9153 (15)	134
N2—H2*A*···S1^ii^	0.88	2.59	3.4319 (14)	161
N3—H3*B*···S1^iii^	0.88	2.59	3.4540 (15)	169

Symmetry codes: (i) −*x*−1, −*y*+1, −*z*; (ii) −*x*+2, −*y*+1, −*z*+1; (iii) −*x*+2, −*y*+2, −*z*+1.
